# Quantitative Comparison against Experiments Reveals Imperfections in Force Fields’ Descriptions of POPC–Cholesterol Interactions

**DOI:** 10.1021/acs.jctc.3c00648

**Published:** 2023-08-24

**Authors:** Matti Javanainen, Peter Heftberger, Jesper J. Madsen, Markus S. Miettinen, Georg Pabst, O. H. Samuli Ollila

**Affiliations:** † Institute of Organic Chemistry and Biochemistry, Academy of Sciences of the Czech Republic, 16000 Prague 6, Czech Republic; ‡ Institute of Biotechnology, University of Helsinki, 00790 Helsinki, Finland; § Biophysics, Institute of Molecular Biosciences, NAWI Graz, University of Graz, 8010 Graz, Austria; ∥ Global and Planetary Health, College of Public Health, University of South Florida, Tampa, Florida 33612, United States; ⊥ Center for Global Health and Infectious Diseases Research, College of Public Health, University of South Florida, Tampa, Florida 33612, United States; # Department of Molecular Medicine, Morsani College of Medicine, University of South Florida, Tampa, Florida 33612, United States; ∇ Fachbereich Physik, Freie Universität Berlin, 14195 Berlin, Germany; ○ Department of Chemistry, University of Bergen, 5007 Bergen, Norway; ◆ Computational Biology Unit, Department of Informatics, University of Bergen, 5008 Bergen, Norway; ¶ BioTechMed-Graz, 8010 Graz, Austria; †† Field of Excellence BioHealth—University of Graz, 8010 Graz, Austria; ‡‡ VTT Technical Research Centre of Finland, 02150 Espoo, Finland

## Abstract

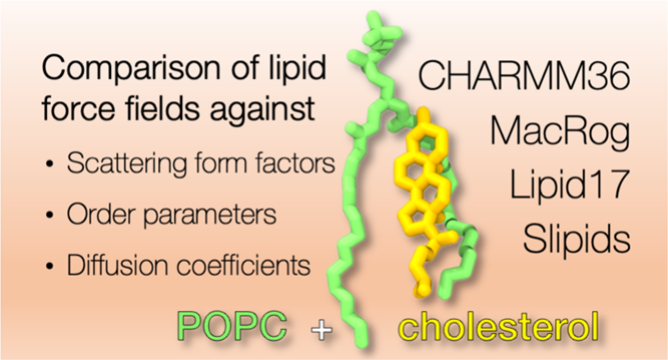

Cholesterol is a central building block in biomembranes, where it induces orientational order, slows diffusion, renders the membrane stiffer, and drives domain formation. Molecular dynamics (MD) simulations have played a crucial role in resolving these effects at the molecular level; yet, it has recently become evident that different MD force fields predict quantitatively different behavior. Although easily neglected, identifying such limitations is increasingly important as the field rapidly progresses toward simulations of complex membranes mimicking the in vivo conditions: pertinent multicomponent simulations must capture accurately the interactions between their fundamental building blocks, such as phospholipids and cholesterol. Here, we define quantitative quality measures for simulations of binary lipid mixtures in membranes against the C–H bond order parameters and lateral diffusion coefficients from NMR spectroscopy as well as the form factors from X-ray scattering. Based on these measures, we perform a systematic evaluation of the ability of commonly used force fields to describe the structure and dynamics of binary mixtures of palmitoyloleoylphosphatidylcholine (POPC) and cholesterol. None of the tested force fields clearly outperforms the others across the tested properties and conditions. Still, the Slipids parameters provide the best overall performance in our tests, especially when dynamic properties are included in the evaluation. The quality evaluation metrics introduced in this work will, particularly, foster future force field development and refinement for multicomponent membranes using automated approaches.

## 1 Introduction

Cellular membranes contain an incredibly complex mixture of lipid molecules^1^ unevenly distributed in the membrane plane and across its leaflets.^2−4^ A key player driving the lateral heterogeneity is cholesterol (CHOL), which is present at concentrations from ∼10 mol % (endoplasmic reticulum) up to ∼50 mol % (plasma membrane, viral envelopes).^2^ CHOL has the unique ability to order neighboring lipids and thus induce the liquid-ordered (L_o_) phase in model membranes.^5−8^ In the cellular setting, the interaction between other lipids and CHOL is associated with the formation of lipid rafts and nanodomains.^9,10^ This heterogeneity can then further regulate protein distribution^11^ or conformation,^12^ in addition to the direct modulation of protein function.^13−15^

While the structure and dynamics of heterogeneous membranes are difficult to capture experimentally, atom-resolution molecular dynamics (MD) simulations have been employed to obtain a detailed view of the lateral organization driven by lipid–CHOL interactions.^8,16−19^ Further MD efforts are facilitated by the growing availability of force fields with compatible lipid and protein parameters—enabling simulations of ever more complex and thus biologically relevant membranes.^1^

The traditional protein force fields CHARMM,^20^ AMBER,^21^ and OPLS^22,23^ now have sizable libraries of compatible lipid molecules, including CHOL, in the forms of CHARMM36,^24,25^ Lipid17/Slipids,^26−31^ and the force field by Maciejewski and Róg (here “MacRog”),^32−35^ respectively. Notably, simulations using CHARMM36, Lipid17, and Slipids can these days be set up for multiple simulation engines with the CHARMM-GUI online tool; this decreases greatly the manual work needed to run complex membrane simulations.^36,37^

While simulating complex membranes with CHOL has become a relatively straightforward task, estimating the trustworthiness of MD simulations remains a challenge, especially as the complexity and number of membrane components increase. Our earlier work has demonstrated that the conformational ensembles of lipids from an MD simulation can be evaluated against the C–H bond order parameters from NMR experiments.^38−42^ This approach has been useful for finding the best force fields to describe the headgroups of phosphatidylcholine (PC),^38,43^ phosphatidylserine (PS),^41^ phoshatidylethanolamine (PE),^42^ and phosphatidylglycerol (PG) lipids;^42^ to evaluate and improve membrane interactions with ions^40−42,44,45^ and small molecules;^46^ and to find simulation parameters predicting most realistic packing properties of membranes.^47,48^ Furthermore, quantitative quality measures based on the C–H bond order parameters have been recently defined and used to rank simulations in the NMRlipids Databank.^48^ However, such automatic quality evaluation is limited to simulations for which experimental data at the corresponding composition and temperature are available. Because simulations mimicking all experimental compositions for multicomponent membranes are often tedious to produce, quality evaluation of mixed lipid bilayers is not yet fully automatized in the NMRlipids Databank.

Here, we demonstrate how simulations of binary palmitoyloleoylphosphatidylcholine–cholesterol (POPC–CHOL) mixtures can be evaluated against experimental NMR spectroscopy and X-ray scattering data by interpolating through multiple CHOL concentrations. As the effect of CHOL on lipid headgroup (and its independence from acyl chain ordering) has been discussed previously,^38,49^ we focus here on the acyl chains; these are expected to play a larger role than the headgroup in CHOL-induced lateral membrane heterogeneity. We also evaluate the dependence of lateral diffusion coefficients on CHOL against pulsed field gradient (PFG) NMR experiments^50,51^ using the recent theoretical framework that allows a quantitative comparison with experiment by eliminating finite-size effects in MD simulations.^52,53^ With the structural and dynamic comparisons established, we then estimate the quality of the four popular force fields at different CHOL concentrations. While we focus here on a POPC–CHOL mixture, we expect our results to set guidelines for future efforts to validate intermolecular interactions for other phosphatidylcholine–cholesterol mixtures, as well as any binary or multicomponent systems.

## 2 Methods

### 2.1 X-ray Scattering Experiments

Fully hydrated multilamellar vesicles (MLVs), composed of POPC and CHOL with the latter present at 0–50 mol % with 5 mol % increments, were prepared for small-angle X-ray scattering (SAXS) experiments using rapid solvent exchange as described previously.^54,55^ This avoids the precipitation of CHOL crystallites at high concentrations,^56^ yielding non-phase-separated samples up to 50 mol % CHOL content. Lipids, purchased from Avanti Polar Lipids (Alabaster, Alabama), were used as dry powders without any further purification. All other chemicals were obtained in pro analysis quality from Lactan (Graz, Austria). The data were obtained at the EMBL BioSAXS beamline (Hamburg) using 20 keV photons at *T* = 300 K and analyzed in terms of the scattering density profile (SDP)-GAP model described in refs (57) and (58). The data from MLVs contain the structure factor (the crystalline lattice) and form factor in a convoluted fashion, yet by fitting the scattered intensity data, we obtained both contributions. The electron density profiles were modeled from form factors using the SDP model, where volume distribution functions are described by individual Gaussians or error functions.^59−61^ CHOL was described using two Gaussians, as detailed in refs (55) and (62). The membrane thickness was defined as twice the distance from the electron density maximum to the membrane center. The electron density maxima were extracted using the findpeaks function in Matlab. The X-ray scattering data as well as the order parameter data from NMR are available at https://github.com/NMRLipids/NmrLipidsCholXray (DOI: 10.5281/zenodo.8172173). These data are also added in the NMRlipids databank (https://github.com/NMRLipids/Databank/tree/main/Data/experiments, DOI: 10.5281/zenodo.7875567), and are available using the NMRlipids protocols described in ref (48).

### 2.2 Molecular Dynamics Simulations

We performed MD simulations of a pure POPC membrane as well as five POPC/CHOL mixtures with CHOL content ranging from 11 to 47 mol % (Table 1). Systems were simulated using four commonly used force fields: CHARMM36 (often abbreviated “C36” in figure legends in this work),^24,25^ Amber-compatible Slipids^28−30^ with its 2020 update,^31^ Amber-compatible Lipid17,^26,27^ and all-atom-OPLS-compatible MacRog.^33−35^ To eliminate the finite-size effects due to periodic boundary conditions from lateral diffusion coefficients of lipids, we performed all simulations in three sizes (64, 256, or 1024 POPC molecules). The number of POPC molecules was kept constant across the different CHOL concentrations. All membranes were solvated by 50 water molecules per lipid (POPC or CHOL). The small membranes (with 64 lipids) were first generated using CHARMM-GUI and equilibrated using the standard protocols for CHARMM36, Slipids, and Lipid17, for which inputs are readily available from CHARMM-GUI.^36,37^ Then, the atomic coordinates were replicated in the membrane plane to create the 4- and 16-fold larger simulation systems (Table 1). Since CHARMM-GUI does not support MacRog, the production simulations were initiated from equilibrated CHARMM36 structures since the two force fields share the atom ordering. All simulations were 1 μs long, totaling 72 μs. The first 10 ns of each simulation was omitted from analyses. The simulation parameters are provided in Table S1, and the simulation data are available at DOI: 10.5281/zenodo.7035350 (CHARMM36), DOI: 10.5281/zenodo.7022749 (Slipids), DOI: 10.5281/zenodo.6992065 (Lipid17), and DOI: 10.5281/zenodo.7061800 (MacRog). All simulations were added to the NMRlipids Databank^48^ and their ID numbers are listed in Table S2. These ID numbers can be used to access the raw simulation data and experimental data linked to each simulation using the NMRlipids protocols described in ref (48). The CHARMM36 simulations have been previously analyzed for their dynamic properties in ref (63).

We used the widely popular and flexible GROMACS simulation engine (version 2020 or 2021)^64^ which enables simulations with a wide range of different force field parameters. While the MacRog and Slipids models were originally parametrized using GROMACS, the parameters for CHARMM36 and Lipid17 were optimized using other simulation engines. This might lead to small differences in behavior due to the differences in algorithm implementation, and sometimes the same algorithms are not available in all simulation engines.^36^ However, the evaluation of differences in force field behavior between different simulation engines is beyond the scope of this work.

**Table 1:** Details of the (Small/Medium/Large) Simulation Systems^a^

[CHOL] (mol %)	POPC	CHOL	water	*x* and *y* (nm)	*z* (nm)
0	64/256/1024	0/0/0	3200/12 800/51 200	4.4/9.0/18.1	8.9/8.6/8.5
11	64/256/1024	8/32/128	3600/14 400/57 600	4.5/9.4/18.1	9.4/9.1/9.3
20	64/256/1024	16/64/256	4000/16 000/64 000	4.5/9.2/18.3	10.2/9.9/10.0
29	64/256/1024	26/104/416	4500/18 000/72 000	4.6/9.2/18.5	10.8/10.8/10.7
38	64/256/1024	40/160/640	5200/20 800/83 200	4.8/9.5/19.2	11.3/11.4/11.2
47	64/256/1024	56/224/896	6000/24 000/96 000	5.0/10.1/20.0	11.8/11.6/11.7

a The box dimensions in the membrane plane (*x* and *y*) and normal to the membrane (*z*) are provided for the Slipids simulations; the values vary slightly between the force fields.

### 2.3 Simulation Analyses

#### 2.3.1 Structural Properties

The C–H bond order parameters, form factors, and electron density profiles, automatically calculated by the NMRlipids Databank,^48^ were used. Similarly to the experimental X-ray scattering data, the membrane thickness was defined as twice the distance from the electron density maximum to the membrane center. Locations of maxima were extracted by the findpeaks function in Matlab after smoothening the electron density data with the smooth function (5-point moving interval) in Matlab. Area per phospholipid was obtained by dividing the area of the bilayer (area of the simulation box) by the number of phospholipids in one leaflet.

To simplify the interpolation of order parameter data to intermediate CHOL concentrations (see “Quantitative quality evaluation of the effect of CHOL on membrane properties” below), C–H bond order parameters of the 2 (3) hydrogens in CH_2_ (CH_3_) groups in the POPC acyl chains were averaged. These groups rotate freely and thus the order parameters are essentially identical for both (all) hydrogens in experiments and simulations. An exception to this are the hydrogens bound to the second carbon in the oleate chain; they lack rotational averaging in both simulations and experiments and were thus treated separately in our analyses. The C–H order parameters for the POPC headgroup are shown separately for all CH_2_ groups, i.e., no averaging was performed.

#### 2.3.2 Lateral Diffusion Coefficients

Lateral diffusion coefficients *D*_PBC_ from simulations performed using periodic boundary conditions (PBC) were extracted from mean squared displacement (MSD) data calculated for lipid centers of mass after the drift of their host leaflet was eliminated with the gmx msd tool. The MSD data were fit with a straight line in the lag time (Δ) interval between 10 and 100 ns as
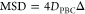
1

The diffusion coefficients extracted from the three simulation box sizes (and thus membrane edge lengths *L*) were fitted with
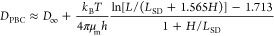
2where *D*_∞_ is the lateral diffusion coefficient in an infinite system, *h* is the hydrodynamic thickness of the membrane, *k*_B_ is the Boltzmann constant, *T* is the temperature, *H* is half the thickness of the water layer,  is the Saffman–Delbrück length, and μ_m_ and μ_f_ are shear viscosities of the membrane and the fluid (water), respectively.^53^ Thus, the fit of eq 2 to the *D*_PBC_ values calculated from the simulation as a function of simulation box size *L* has two free parameters, namely, *D*_∞_ and μ_m_, both of which are free of finite-size effects and can thus be compared to experiment. The interleaflet friction coefficient does not appear in eq 2 as we expect it to be infinite, which was found to be a valid assumption for lipid bilayers.^53^ The water viscosity value of μ_f_ = 0.3228 mPa s was interpolated to 298 K from the values for CHARMM TIP3P in ref (65) and used for all simulations (CHARMM TIP3P and normal TIP3P differ by ∼2 to 3%). The simulation box dimensions (membrane edge length *L* and dimension normal to the membrane plane (*z*) *L*_*z*_) were taken from the final configuration of each simulation. *L*_*z*_ was needed only in the calculation of *H* (see below). Membrane thickness *h* was obtained as described in “Structural properties” above. As *h* and *H* = (*L*_*z*_ – *h*)/2 are constants in eq 2, the average values from the three systems sizes were used in the fits.

#### 2.3.3 Quantitative Quality Evaluation of the Effect of CHOL on Membrane Properties

To ease the evaluation of simulations against experimental data with nonmatching CHOL concentrations, we interpolated the effect of CHOL in simulations and experiments for CHOL concentrations ranging from 0 to 46 mol %. Two-dimensional (2D) matrices were created by interpolation for the oleate and palmitate chain order parameters (as a function of carbon atoms in the acyl chains and CHOL concentration) using the interp2 function in Matlab based on linear interpolation. Similar matrices were also generated for the electron density profiles (as a function of distance from bilayer center and CHOL concentration) but with the scatteredInterpolant function in Matlab based on Delaunay triangulation.^66^ Linear 1D interpolation with the interp1 function in Matlab was used for the CHOL dependence of the first and second form factor minima locations and diffusion coefficients. These interpolations were then used to calculate deviations (in %) from experimental values across CHOL concentrations to quantify the quality of the lipid force fields. For the 2D matrices, the absolute values of the differences between the matrices from simulations and experiments were first calculated. The averages of differences over the carbon atoms in the acyl chain (order parameters) or across the entire *z* coordinate in the simulation (density profiles) were then calculated. This resulted in 1D deviation vectors as a function of the CHOL concentration. For diffusion coefficients and form factor minima (strictly speaking: minima of the absolute value of the form factor), the absolute value of the difference of the interpolated 1D vectors of simulation and experimental data was calculated to provide deviation as a function of CHOL concentration. All of these 1D vectors were normalized by dividing them by the experimental values to provide relative deviations in % between a simulation and experiments as a function of CHOL concentration. For C–H bond order parameters, the deviation matrices between simulations and experiments were also used to illustrate the quality of simulations.

## 3 Results and Discussion

### 3.1 Acyl Chain Ordering Varies Greatly between the Force Fields

CHOL is known to induce order in lipid membranes by increasing the fraction of anti conformations in the acyl chains of phospholipids,^67^ which is suggested to play a critical role in the phase behavior of PC–CHOL mixtures.^6^ Consequently, the correct CHOL-induced ordering is expected to be a necessary condition for a force field used to understand lipid–CHOL phase behavior. The CHOL-induced ordering can be experimentally quantified by measuring the C–H bond order parameters using ^13^C or ^2^H NMR, and the results can also be directly compared to MD simulations.^39^ Simulations generally show some CHOL-induced ordering, but the order parameter values often deviate from the experimental ones at high CHOL concentrations.^27,67^ Furthermore, it has not been clear how accurately different force fields capture the details of lipid–CHOL interactions and which force field would give most realistic results for simulations of complex mixtures, where such interactions play critical roles.

**Figure 1:**
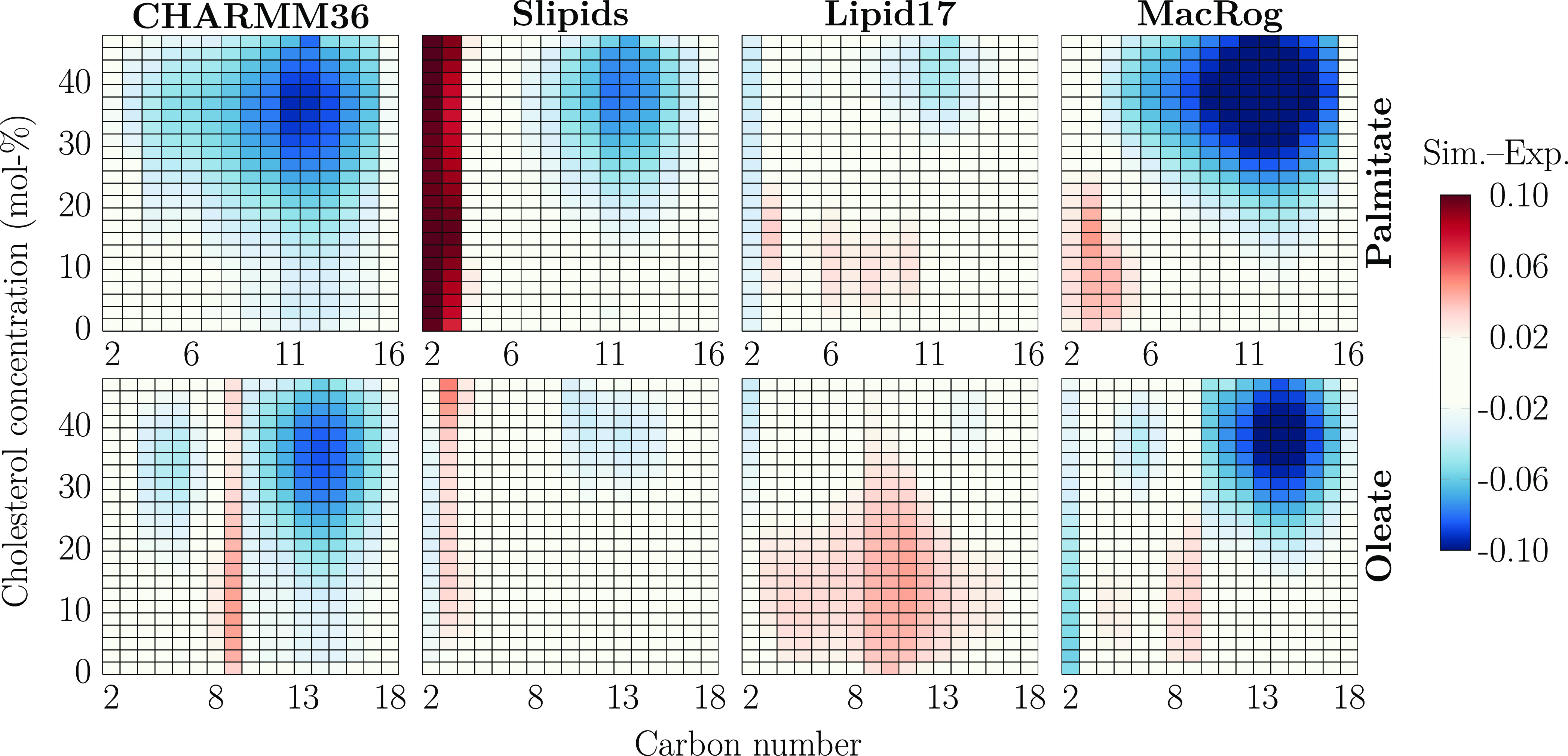
POPC acyl chain order parameter deviation from experiments. Data are shown for palmitate (top row) and oleate (bottom row) and for the four force fields (columns). Negative values indicate that the order is too high (*S*_CH_ values are too negative) in the simulations. The values that are within the estimated experimental error range of ±0.02^39^ are colored white. Statistical error in simulations is not considered here because it is approximately an order of magnitude smaller than the experimental error. Order parameters of hydrogens attached to the same carbon were averaged, except for the C2 carbon of the oleate chain (whose order parameters were forked), for which differences for both the larger and smaller values were calculated, and the average of these differences is shown in the C2 column.

Here, we evaluate the CHOL-induced ordering in state-of-the-art force fields against C–H bond order parameter data from ^13^C NMR experiments measured from POPC–CHOL mixtures with CHOL concentrations ranging between 0 and 60 mol %.^67^ To this end, we first interpolated order parameter maps as a function of the acyl chain carbon number and CHOL concentration for both simulations and experiments. These maps were then subtracted to obtain deviation maps between the simulations and experiments. The deviation maps of different force fields are shown for the palmitate (top row) and oleate (bottom row) chains of POPC in Figure 1, whereas the original order parameter profiles are shown in Figures S6 and S7 for the palmitate and oleate chains, respectively.

In the simulations with all tested force fields as well as in experiments, the CHOL-induced ordering is manifested in the original profiles (Figures S6 and S7) as a substantial increase in the absolute values of acyl chain C–H bond order parameters upon addition of CHOL. The deviations mapped in Figure 1 provide an intuitive view for a quantitative comparison of different force fields against experiments: In white regions, the simulation results are considered to fall within the experimental error as the deviations are in the range of [−0.02,0.02]; blue indicates that the order parameters are too negative; i.e., the acyl chains are too ordered in simulations, and vice versa for red. Overall, the simulation force fields behave reasonably well at low CHOL concentrations but deviate significantly from experiment at higher CHOL concentrations.

In CHARMM36, both the palmitate and oleate chains become too ordered upon increasing CHOL concentration. The oleate chain shows the best agreement with experiment in Slipids, whereas there is some excess ordering in the palmitate chain. Still, the major discrepancy between Slipids and the experiment is the drastically too disordered C2 and C3 carbons of the palmitate chain. This effect was introduced in the recent reparametrization of Slipids that improved the headgroup and glycerol backbone structures of Slipids.^31^ Lipid17 provides the best overall agreement with experiment, as no segments deviate significantly from experiment at any CHOL concentrations. MacRog behaves reasonably well at low CHOL concentrations, yet at larger CHOL concentrations, the chains become too ordered, leading to the largest overall deviations from experiment. For the headgroup and glycerol backbone order parameters, provided in Figure S8, CHARMM36 gives the best agreement at all cholesterol concentrations, in line with previous studies.^38,49^

### 3.2 Cholesterol Effect on Membrane Properties Manifests Differently in Different Force Fields

CHOL-induced ordering straightens the acyl chains, which leads to membrane thickening. While acyl chain order and membrane thickness are well correlated,^48^ lipid bilayer dimensions can be accessed more directly by measuring the X-ray form factor, which is related to the electron density along membrane normal via Fourier transform.^39,58,68,69^ Electron density profile, area per phospholipid, and bilayer thickness can all be extracted from the form factor using the scattering density profile (SDP) model or its combination with MD simulations.^58,60,68−70^ To complement the evaluation of CHOL-induced ordering against NMR order parameters, we also measured the X-ray form factors from POPC–CHOL mixtures at systematically increasing CHOL concentrations. Scattering intensities from experiments are shown in Figures S1 and S2, form factors from experiments and simulations in Figure S3, and density profiles in Figure S4.

**Figure 2:**
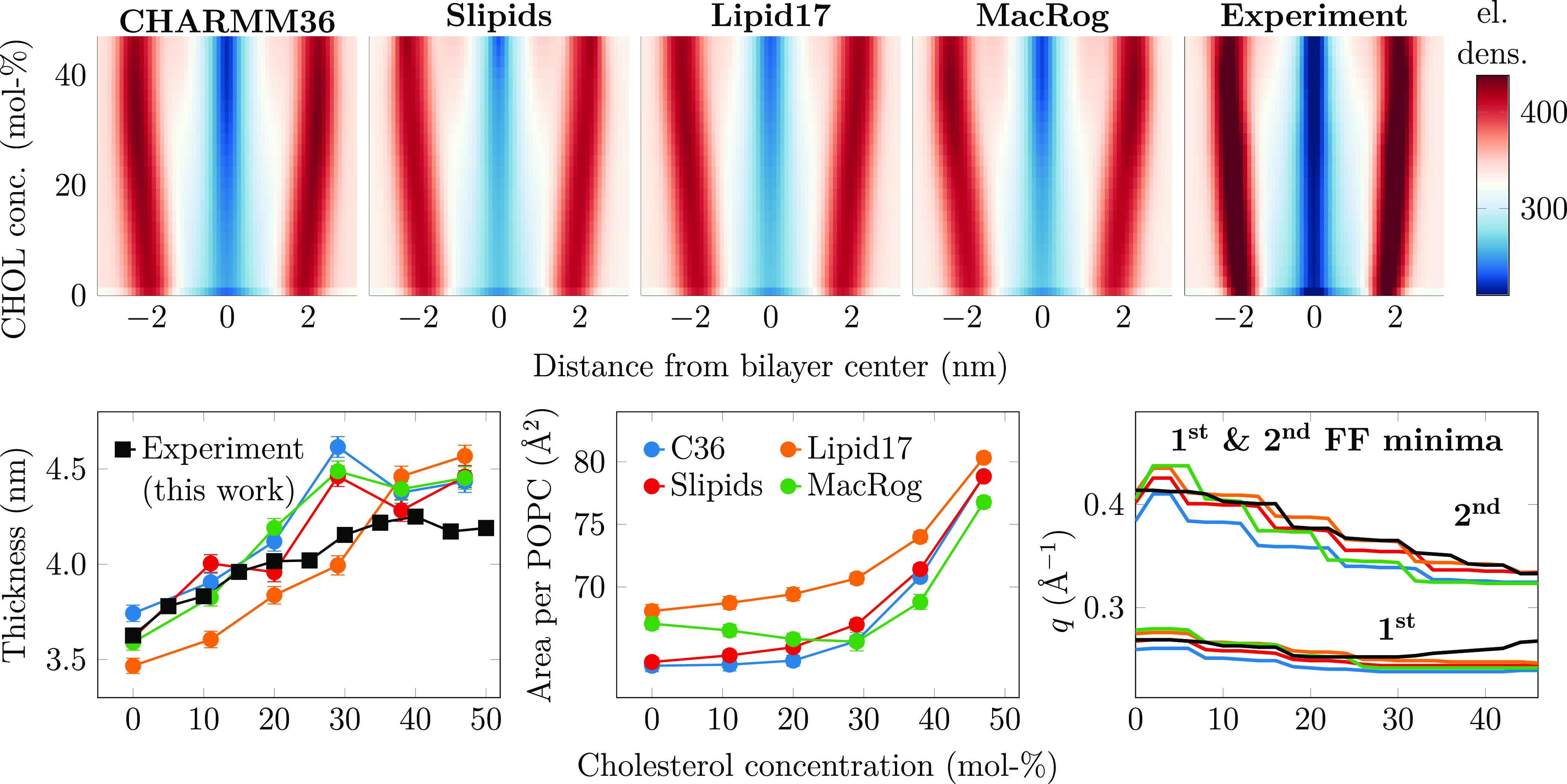
Electron density profiles, thickness, and area per phospholipid as a function of CHOL concentration. Top: electron density maps were created for the simulations using four different force fields and for the experiment (from the SDP model). The color bar is common for all maps. The original electron density profiles are shown in Figure S4. The effect of system size on the density profiles in simulations is demonstrated in Figure S13 in the Supporting Information. Bottom left: bilayer thickness. Thickness is defined as twice the distance from the peak in electron density to the membrane core. Experimental data are extracted in a similar manner from electron density profiles obtained with X-ray scattering. The bin width used in the profiles is used as the error estimate. Bottom middle: area per phospholipid measured by dividing the total membrane area by the number of phospholipids. Error bars show standard error estimated using block averaging implemented in gmx analyze of GROMACS. The size dependency of the area per phospholipid is shown in Figure S12. Bottom right: effect of CHOL on the location of the first two minima in the form factor. The minima are extracted from the form factors interpolated to all CHOL concentrations (Figure S3) from experiment and simulation with the findpeaks function in Matlab. Because differences between experiments and simulations for the first minimum location are barely visible for some force fields, we have highlighted the relative deviations in Figure S5.

The effect of CHOL on the structural properties of bilayers is compared between the SDP model (based on experimental form factors) and MD simulation results in Figure 2. All force fields demonstrate increasing thickness upon the addition of CHOL that tends to saturate after approximately 30 mol % (bottom middle panel in Figure 2). Most MD simulations agree well with the SDP model below 30 mol % but overshoot the SDP results at high CHOL concentrations. Lipid17 simulations are the exception: They predict thinner membranes than the SDP model at low CHOL concentrations, and a clear saturation of thickness is not observed, unlike for other force fields and experiments.

The dependence of the area per phospholipid (APL) on CHOL concentration follows the trends in thickness inversely in general (bottom right panel of Figure 2), yet provides curious differences between force fields at the physiologically relevant CHOL concentration range.^2^ Lipid17 has the largest APL across the entire CHOL concentration range. MacRog also has a large APL for pure POPC, but the partial area of CHOL is negative until 30 mol % concentration, indicating a particularly strong condensation effect. The profiles for Slipids and CHARMM36 are very similar, with a small or zero partial area of CHOL until a concentration of 20 mol %.

For a more detailed comparison of membrane structure, we interpolated the changes in electron density profiles along the membrane normal (Figure S4) as a function of CHOL concentration to create two-dimensional electron density maps (Figure 2). Overall, all electron density profiles share the same features across all CHOL concentrations: High densities corresponding to the tightly packed interfacial regions containing the electron-rich phosphorus, low density at the core of the membrane occupied by the disordered acyl chains, and intermediate density in the rest of the lipid regions as well as the aqueous phase. However, a more detailed look at the profiles in Figure 2 reveals differences between the force fields and the SPD model. CHARMM36 has the sharpest low- and high-density bands among MD simulation results, indicating smaller membrane undulations that would smear the bands; the same is true for MacRog at higher CHOL concentrations. The less sharp bands for Lipid17 and especially Slipids profiles indicate that their membranes are more flexible. The SDP model gives the sharpest bands (top right panel in Figure 2 and original electron density profiles in Figure S4), suggesting more uniform membrane structure than any MD simulation. However, system size plays a role here as undulations smear out the density profile features with increasing simulation box size, as demonstrated in Figure S13 by the density maps calculated for the CHARMM36 simulations in three sizes. Still, even in the smallest system, the band intensities are less localized than those in the SDP model. This might indicate different elastic properties between simulation and experiment, but we cannot fully exclude the role of models used to interpret the scattering data.

Also the form factor profiles depend on simulation system size (see Figure S2 of ref 48) which complicates their direct comparison with experimental data. Nevertheless, the minima in X-ray form factors are independent of the simulation box size and correlate with membrane dimensions.^48^ To avoid the effects from simulation system sizes, we thus interpolated the locations of the first two minima in the form factors over the entire studied range of CHOL concentrations (bottom right panel of Figure 2) for a more direct comparison between simulations and experiments. These curves highlight that at first the addition of CHOL shifts the first minima to smaller wave vector values in the experiment; this is reasonably well captured by the simulation force fields, although CHARMM36 seems to be off more than the other three force fields. Above ∼25 mol % CHOL, the location of the minimum shifts to larger wave vector values in the experiment, which is curiously not captured by any of the force fields. For the second minimum, the experiment shows a steady shift to smaller wave vector values; this is reproduced by all simulation force fields, but Slipids and Lipid17 are generally in better agreement with the experiment than MacRog and CHARMM36.

### 3.3 Force Fields Predict Very Different Lateral Mobilities

**Figure 3:**
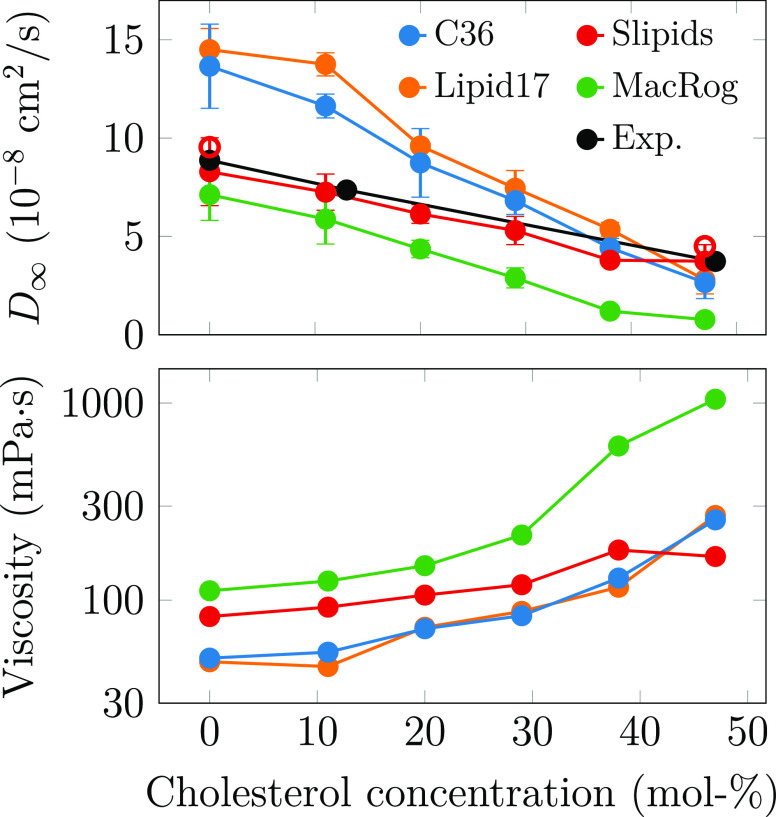
Dynamic properties of the POPC/CHOL mixtures. Top: POPC lateral diffusion coefficients were corrected for finite-size effects using eq 2. Experimental data are taken from NMR measurements of well-hydrated samples.^50,51^ The hollow circles show data extracted for Slipids using a shorter Lennard-Jones cutoff (see text). The error bars show the standard deviations of the diffusion coefficients obtained from 10,000 fits to the values *D* ± Δ*D*, where Δ*D* are sampled from normal distributions whose standard deviations are equal to the error estimates of the corresponding values of *D*. For these error estimates, we used the differences in the diffusion coefficients extracted from the two membrane leaflets. Bottom: shear viscosities obtained from the finite-size correction, eq 2. The distributions of the viscosity values from the 10,000 fits (see above) were often skewed and thus could not be fitted by a single Gaussian, complicating the error estimation. The results from double-Gaussian fits to the data are presented in Figure S11. The size dependence of lateral diffusion and the fits used to obtain the PBC-corrected diffusion coefficients and shear viscosities are shown in Figures S9 and S10.

Apart from the ordering effect on the bilayer structure, CHOL is also known to make bilayers stiffer and less dynamic.^8,50,51^ The comparison between lateral diffusion coefficients extracted from simulation and experiment has been limited due to a box size dependence observed in simulations performed using periodic boundary conditions (PBCs).^52,53,71^ Here, we tackle this issue by performing simulations with three system sizes, which allows extrapolation of the results to an infinite system with the theoretical description developed by Vögele and Hummer.^52,53^ The size dependence of phospholipid lateral diffusion from simulations together with the fit of eq 2 are shown in Figure S9, whereas the CHOL dependencies of these values in systems with different sizes are shown in Figure S10. The PBC-corrected lateral diffusion coefficients of POPC are shown in the top panel of Figure 3 together with experimental values from ^1^H pulsed field gradient NMR diffusion measurements on label-free macroscopically aligned bilayers.^50,51^

The lipid force fields again show significantly different behaviors. Lipid17 and CHARMM36 show too fast dynamics for pure POPC, and the slowdown induced by CHOL is exaggerated compared to experiment. Diffusion in MacRog is generally too slow. In contrast, Slipids provides an essentially quantitative agreement with experiment across the studied CHOL concentrations and thus significantly outperforms the other force fields in terms of lateral dynamics. Interestingly, there is no correlation between the deviations from experiments and the structural properties described earlier. To investigate if the differences in lateral diffusion coefficients could be explained by different Lennard-Jones (LJ) cutoff values, we repeated simulations at 0 and 47 mol % CHOL for Slipids using a shorter cutoff of 0.9 nm (corresponding to that of Lipid17, while original cutoff for Slipids was 1.4 nm, see Table S1). The PBC-corrected diffusion coefficient values with shorter cutoff, shown in the top panel of Figure 3 as hollow red circles, are only slightly larger than the original values, indicating that differences arise from a combination of force field interaction parameters and are not explained by the LJ cutoff alone.

When comparing lipid lateral diffusion between simulations and experiments, it is important to note that the PBC correction not only affects the values of diffusion coefficients but also qualitatively changes their trends as a function of cholesterol. This results from the fact that the size of the PBC correction, eq 2, depends on membrane viscosity, which further depends on CHOL concentration. For example, Figure S10 would indicate that Lipid17 and CHARMM36 systematically underestimate the experimental values in systems with all sizes simulated here, while the effect of CHOL seems to be qualitatively reproduced. However, their PBC-corrected values significantly overshoot the experiment at low CHOL concentration, and CHOL induces a more drastic slowdown in simulations, leading to values close to experimental ones at 47 mol % CHOL. With MacRog, also the PBC-corrected values fall below the experimental ones, yet the slowdown effect of CHOL still appears stronger than in experiment. With Slipids, the CHOL dependence seems too weak with finite system sizes, yet after accounting for PBC effects, the agreement with the experiment is excellent. These results underline that little can be said about the CHOL dependence of lateral diffusion coefficients without the PBC correction that requires either performing simulations with multiple box sizes at different CHOL concentrations or estimating the membrane viscosity for eq 2 in some other way.^72^ Consequently, fine tuning of interaction parameters to correctly reproduce lateral diffusion coefficients can require a massive number of simulations.

We also provide the shear viscosity values of the membranes obtained from the fits of eq 2 in Figure 3. Direct comparison of these to experimental values is complicated due to the large scatter of experimental estimates,^73^ yet values from Slipids are expected to be most realistic because its lateral diffusion coefficients are closest to experiments. Note that the viscosities from CHARMM36 simulations are further discussed in our previous work.^63^

### 3.4 Quality of Force Fields at Various Cholesterol Concentrations

**Figure 4:**
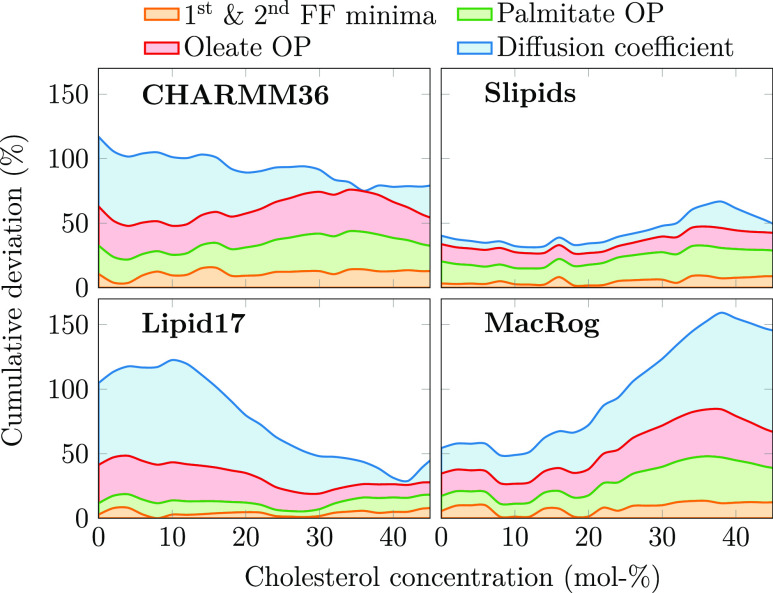
Total relative deviation of force fields from experimental data. Two leftmost columns: the relative deviations in the first two form factor minima, POPC palmitate and oleate chain order parameters, and diffusion coefficients are shown in a cumulative manner to highlight the overall deviation of the force fields from experimental data. The order parameter deviations are obtained by averaging over the columns in Figure 1 and normalizing against experimental data. For the form factor minima (shown at the bottom right in Figure 2), the deviation was obtained by calculating the difference between experiment and simulation for both minima, normalizing each against experimental data, and summing the two together. The diffusion coefficient deviation is the difference of values from simulation and experiment in Figure 3, taken after interpolation to the same CHOL values as shown in Figures 1 and 2, and normalized against experimental values.

To streamline the selection of force fields that best capture the effect of CHOL on the different properties of POPC membranes, we defined quantitative quality measures for simulations. For this, we calculated for all of the force fields their relative deviations from experiments (difference between simulated and experimental values divided by the experimental value) using interpolated data for the form factor minima, the order parameters of the two acyl chains, and the diffusion coefficients. The quality evaluations are shown in Figure 4.

The quality evaluations reveal that Slipids provides the overall best agreement with experiment, yet its quality decreases slightly at higher CHOL concentrations. Lipid17 provides a slightly better agreement with experiment above ∼30 mol % CHOL than Slipids but exhibits major deviation from experiments at low CHOL concentrations. MacRog performs relatively well at low CHOL concentrations, but its quality deteriorates significantly upon addition of CHOL. The deviation for CHARMM36 is significant at all CHOL concentrations.

## 4 Conclusions

The tested all-atom force fields captured the most important general effects of CHOL on POPC membrane properties: increased acyl chain ordering, concomitant increase in bilayer thickness, and reduced lateral diffusion rate of phospholipids, i.e., features associated with the liquid-ordered phase. However, a quantitative comparison reveals differences between force fields and their qualities evaluated against NMR and X-ray scattering data. Comparison with NMR order parameters and X-ray scattering form factors proposes that simulations reproduce experimental results until up to 20 mol % CHOL, but overestimate the acyl chain ordering and membrane thickness after further addition of CHOL. An apparent exception to this is the Lipid17 force field, yet its seemingly better agreement with experiments results from compensation of the initially (at low CHOL concentrations) underestimated acyl chain order by the overly strong response to CHOL addition. In conclusion, a unified picture emerging from comparison with NMR and X-ray scattering data suggests that all of the tested force fields overestimate the CHOL-induced ordering effect, particularly above 20 mol % of CHOL. A previously published comparison^67^ suggested the same conclusion for the historically relevant Berger/Höltje force field parameters^74,75^ that were not included in this work.

Notably, no phase separation or domain formation was evident in our simulations. This differs from mixtures containing lipids with saturated acyl chains such as DPPC, which were recently found to contain hexagonally packed substructures.^76,77^ However, such structures seem to only be present at temperatures below the main transition temperature of the lipid with saturated chains.^78^ As the main transition temperature of POPC lies below 273 K, hexagonal packing is not expected, and our findings are consistent with the earlier studies.

Besides the CHOL-induced ordering, effects on membrane properties have been discussed also in terms of (i) the CHOL condensing effect, which refers to a decrease in the area per phospholipid upon the addition of CHOL (negative partial area),^79^ or (ii) CHOL having a diminishing partial area, meaning that a certain amount of CHOL could be added to a phospholipid bilayer without effecting its total area (zero partial area).^80^ At the physiological CHOL concentrations in the range from 0 to 30 mol %, only MacRog predicts negative partial area for CHOL, while CHARMM36 predicts zero partial area, and Slipids and Lipid17 predict small positive partial areas. Considering that Slipids and Lipid17 perform best in our quality evaluation against experiments, yet still slightly overestimating the CHOL condensing effect, our results suggest that CHOL has a positive but small partial area.

Considering also lateral dynamics and previous evaluation of rotational dynamics against NMR data,^81^ Slipids is overall closest to experiments among the parameters tested here, and is therefore probably the best choice for studies where phospholipid–CHOL interactions play a major role. Nevertheless, all tested parameters capture the qualitative effects of CHOL on POPC membrane properties relatively well and differences between force fields are clearly smaller than, for example, in the case of PC–PS lipid mixtures.^47^ Therefore, the quality of the force field selected for other molecules, such as proteins, sugars, drugs, or lipids other than phosphatidylcholine, and the force field compatibility might be more relevant decisive factors for simulations of complex systems. Finally, it must be noted that while all simulations were performed with their suggested simulation parameters, the different simulation engines might provide slightly different behavior.^36^ This is especially true for the Lipid17 and CHARMM36 force fields, which were originally not parametrized using GROMACS. For example, the overcondensation observed for CHARMM36 in this work resembles earlier findings on the differences between simulation engines for CHOL-free membranes.^36^ We used GROMACS here for all simulations due to its popularity and compatibility with all tested force fields. Unfortunately, a comparison of results obtained with different simulation engines is beyond the scope of this work. Moreover, since the observed differences probably emerge from differences in the handling of Lennard-Jones cutoffs, they are likely to be eliminated in the near future by the emergence of force fields benefiting from LJ-PME.^82−85^

Our results demonstrate that quality evaluation of lipid mixture simulations against experimental NMR and X-ray scattering data gives consistent results for how accurately force field parameters capture intermolecular interactions. The interpolation approach introduced here extends the NMRlipids Databank quality metrics^48^ beyond individual systems: this enables the automatic ranking of not only lipid mixtures but also membranes mixed with other molecules such as ions. Such tools will strongly support the emerging endeavors for automatic improvement of force field parameters.^47^

## Supporting Information Available

Simulation parameters used for all of the studied force fields; form factor plots from simulations and experiments with the traced minima at various cholesterol concentrations; electron density profiles from simulation and experiment at various cholesterol concentrations; C–H order parameters of both acyl chains of POPC from simulation and experiment at various cholesterol concentrations; deviation of POPC headgroup order parameters from experiment as a function of cholesterol concentration; lateral diffusion coefficients with varying cholesterol concentrations as a function of simulation box size from simulations together with the extrapolation of these values to an infinite box size; diffusion coefficients as a function of cholesterol concentration from simulations with varying box sizes together with comparison to experiment; effect of simulation box size on the area per phospholipid across the studied cholesterol concentrations; effect of simulation box size on the density profiles across the studied cholesterol concentrations; and cholesterol tilt as a function of cholesterol concentration from simulations (PDF)
