# Supplementary material for: Quantitative Comparison against Experiments Reveals Imperfections in Force Fields’ Descriptions of POPC–Cholesterol Interactions
Source: J Chem Theory Comput. 2023 Aug 24;19(18):6342–52. doi: 10.1021/acs.jctc.3c00648 (PMC10536986; doi:10.1021/acs.jctc.3c00648)
Supplement: Supplementary file 1 — ct3c00648_si_001.pdf [file ct3c00648_si_001.pdf]

Supporting Information: Quantitative Comparison Against Experiments Reveals Imperfections in Force Fields' Descriptions of POPC–Cholesterol Interactions

Matti Javanainen,[*,†,‡] Peter Heftberger,[¶] Jesper J. Madsen,[§,‖,⊥] Markus S. Miettinen,[#,@,△] Georg Pabst,[¶,∇,††] and O. H. Samuli Ollila[*,‡,‡‡]

†*Institute of Organic Chemistry and Biochemistry, Academy of Sciences of the Czech Republic, 16000 Prague 6, Czech Republic*

‡*Institute of Biotechnology, University of Helsinki, 00790 Helsinki, Finland*

¶*Biophysics, Institute of Molecular Biosciences, NAWI Graz, University of Graz, 8010 Graz, Austria*

§*Global and Planetary Health, College of Public Health, University of South Florida, Tampa, Florida, 33612, United States of America*

‖*Center for Global Health and Infectious Diseases Research, College of Public Health, University of South Florida, Tampa, Florida, 33612, United States of America*

⊥*Department of Molecular Medicine, Morsani College of Medicine, University of South Florida, Tampa, Florida, 33612, United States of America*

#*Fachbereich Physik, Freie Universität Berlin, 14195 Berlin, Germany*

@*Department of Chemistry, University of Bergen, 5007 Bergen, Norway*

△*Computational Biology Unit, Department of Informatics, University of Bergen, 5008 Bergen, Norway*

∇*BioTechMed-Graz, 8010 Graz, Austria*

††*Field of Excellence BioHealth—University of Graz, 8010 Graz, Austria*

‡‡*VTT Technical Research Centre of Finland, Espoo, Finland*

E-mail: matti.javanainen@helsinki.fi; samuli.ollila@helsinki.fi

Contents

1 Simulation Details **S-2**

2 Additional Results **S-5**

2.1 Scattering Intensities S-5

2.2 Form Factors & Electron Density Profiles S-7

2.3 Order Parameters S-9

2.4 Dynamic Properties S-12

2.5 Finite-Size Effects S-15

References **S-17**

1 Simulation Details

The force-field-specific simulation parameters used for each force field are listed in Table S1. The parameter input files (`.mdp`) are provided in the Zenodo portal (links in the main text). For all simulations, we used an integration time step of 2 fs and the leap-frog integrator of GROMACS. The isothermal–isobaric (NPT) ensemble was used with a temperature of 298 K and a pressure of 1 bar. All simulations were 1 µs long, and trajectories were written every 1 ns. The P-LINCS constraint algorithm[S1,S2] was used for the bonds noted in Table S1. NMRlipids Databank[S3] ID numbers of simulations are given in Table S2.

Table S1: Simulation parameters used for different force fields.

**Parameter**	**CHARMM36**	**Slipids**	**Lipid17**	**MacRog**
Neighbour list type	Verlet[S4]	Verlet[S4]	Verlet[S4]	Verlet[S4]
Long-range electrostatics	PME[S5,S6]	PME[S5,S6]	PME[S5,S6]	PME[S5,S6]
LJ cut-off	1.2 nm	1.4 nm	0.9 nm	1.0 nm
LJ modifier	Force switch 1.0–1.2 nm	–	–	–
Dispersion correction	–	Energy & pressure[S7]	Energy & pressure[S7]	Energy & pressure[S7]
Thermostat	Nosé–Hoover[S8,S9]	Stochastic rescaling[S10]	Nosé–Hoover[S8,S9]	Stochastic rescaling[S10]
Time constant (T)	1 ps	0.5 ps	1 ps	0.1 ps
Coupling groups	Lipids & water	Lipids & water	Lipids & water	Lipids & water
Barostat	Parrinello–Rahman[S11]	Berendsen[S12]	Parrinello–Rahman[S11]	Parrinello–Rahman[S11]
Coupling type (P)	semi-isotropic	semi-isotropic	semi-isotropic	semi-isotropic
Time constant (P)	5 ps	10 ps	5 ps	4 ps
Compressibility	$4.5{\cdot}10^{-5}$ 1/bar	$4.5{\cdot}10^{-5}$ 1/bar	$4.5{\cdot}10^{-5}$ 1/bar	$4.5{\cdot}10^{-5}$ 1/bar
Constraints	Bonds with H	All bonds	Bonds with H	All bonds
Water model	TIPS3P[S13]	TIP3P[S14]	TIP3P[S14]	TIP3P[S14]
FF Source	CHARMM-GUI	CHARMM-GUI	CHARMM-GUI	Refs. S15 & S16
Parameter source	CHARMM-GUI	Slipids website	CHARMM-GUI	Refs. S15 & S16

Table S2: NMRlipids Databank IDs of performed simulations.

CHARMM36

System	ID
64POPC_3200SOL_298K	678
256POPC_12800SOL_298K	710
1024POPC_51200SOL_298K	701
64POPC_8CHOL_3600SOL_298K	109
256POPC_32CHOL_14400SOL_298K	119
1024POPC_128CHOL_57600SOL_298K	426
64POPC_16CHOL_4000SOL_298K	525
256POPC_64CHOL_16000SOL_298K	72
1024POPC_256CHOL_64000SOL_298K	88
64POPC_26CHOL_4500SOL_298K	393
256POPC_104CHOL_18000SOL_298K	298
1024POPC_416CHOL_72000SOL_298K	412
64POPC_40CHOL_5200SOL_298K	620
256POPC_160CHOL_20800SOL_298K	275
1024POPC_640CHOL_83200SOL_298K	550
64POPC_56CHOL_6000SOL_298K	91
256POPC_224CHOL_24000SOL_298K	166
1024POPC_896CHOL_96000SOL_298K	543

Slipids

System	ID
Slipids_POPC_S	664
Slipids_POPC_M	708
Slipids_POPC_L	696
Slipids_POPC_CHOL11_S	672
Slipids_POPC_CHOL11_M	681
Slipids_POPC_CHOL11_L	697
Slipids_POPC_CHOL20_S	692
Slipids_POPC_CHOL20_M	687
Slipids_POPC_CHOL20_L	691
Slipids_POPC_CHOL29_S	693
Slipids_POPC_CHOL29_M	659
Slipids_POPC_CHOL29_L	709
Slipids_POPC_CHOL38_S	661
Slipids_POPC_CHOL38_M	668
Slipids_POPC_CHOL38_L	670
Slipids_POPC_CHOL47_S	703
Slipids_POPC_CHOL47_M	712
Slipids_POPC_CHOL47_L	682

Lipid17

System	ID
Lipid17_POPC_S	715
Lipid17_POPC_M	657
Lipid17_POPC_L	684
Lipid17_POPC_CHOL11_S	680
Lipid17_POPC_CHOL11_M	689
Lipid17_POPC_CHOL11_L	663
Lipid17_POPC_CHOL20_S	662
Lipid17_POPC_CHOL20_M	666
Lipid17_POPC_CHOL20_L	667
Lipid17_POPC_CHOL29_S	686
Lipid17_POPC_CHOL29_M	700
Lipid17_POPC_CHOL29_L	683
Lipid17_POPC_CHOL38_S	688
Lipid17_POPC_CHOL38_M	694
Lipid17_POPC_CHOL38_L	673
Lipid17_POPC_CHOL47_S	707
Lipid17_POPC_CHOL47_M	714
Lipid17_POPC_CHOL47_L	717

MacRog

System	ID
MacRog_POPC_S	674
MacRog_POPC_M	675
MacRog_POPC_L	658
MacRog_POPC_CHOL11_S	665
MacRog_POPC_CHOL11_M	695
MacRog_POPC_CHOL11_L	660
MacRog_POPC_CHOL20_S	702
MacRog_POPC_CHOL20_M	706
MacRog_POPC_CHOL20_L	677
MacRog_POPC_CHOL29_S	690
MacRog_POPC_CHOL29_M	655
MacRog_POPC_CHOL29_L	705
MacRog_POPC_CHOL38_S	716
MacRog_POPC_CHOL38_M	679
MacRog_POPC_CHOL38_L	698
MacRog_POPC_CHOL47_S	704
MacRog_POPC_CHOL47_M	699
MacRog_POPC_CHOL47_L	671

2 Additional Results

2.1 Scattering Intensities

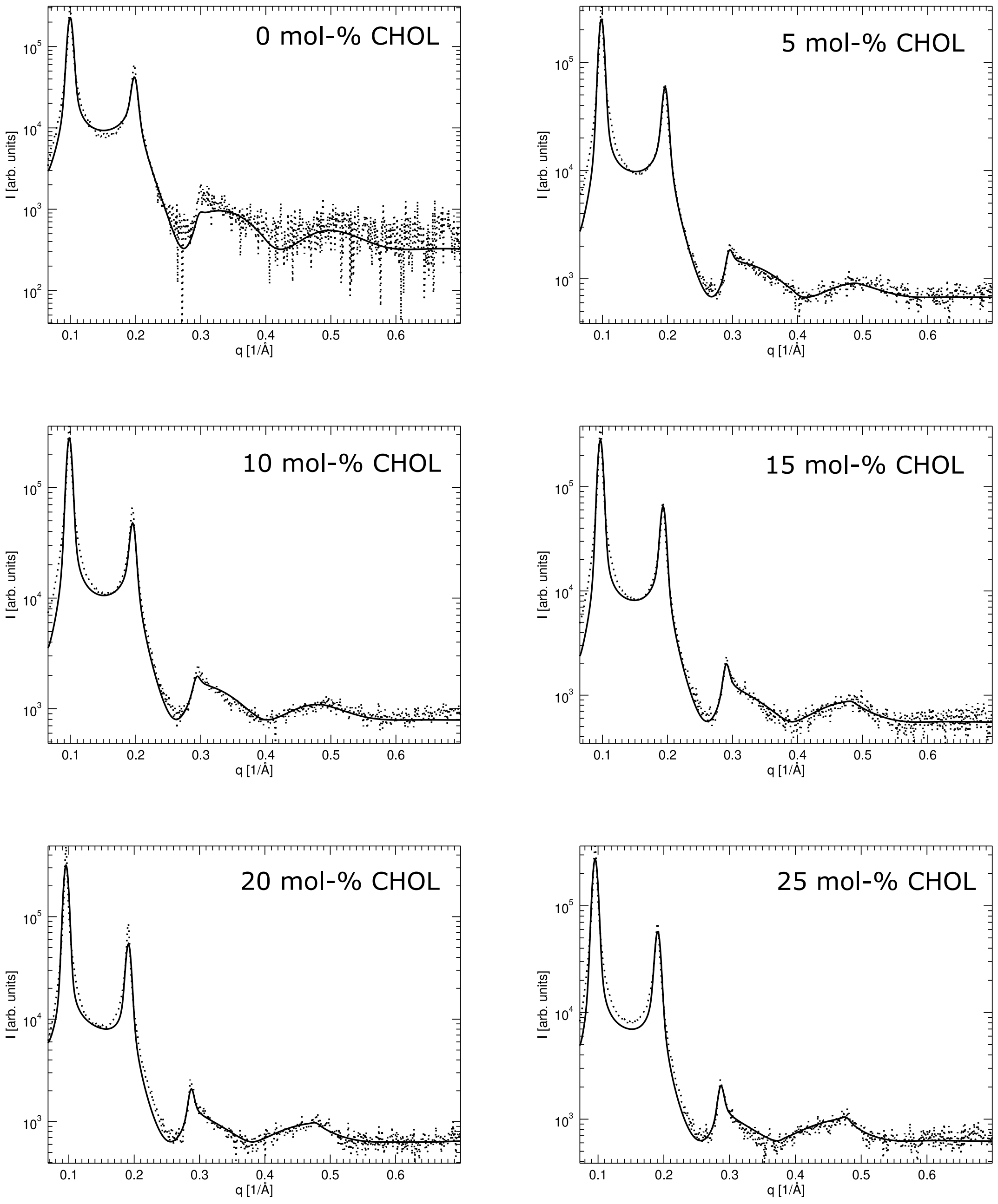

Figure S1: **Scattering intensities from X-ray scattering experiments with various concentrations of cholesterol.** Solid lines correspond to best fits using the SDP-GAP model. More data are shown in the next figure.

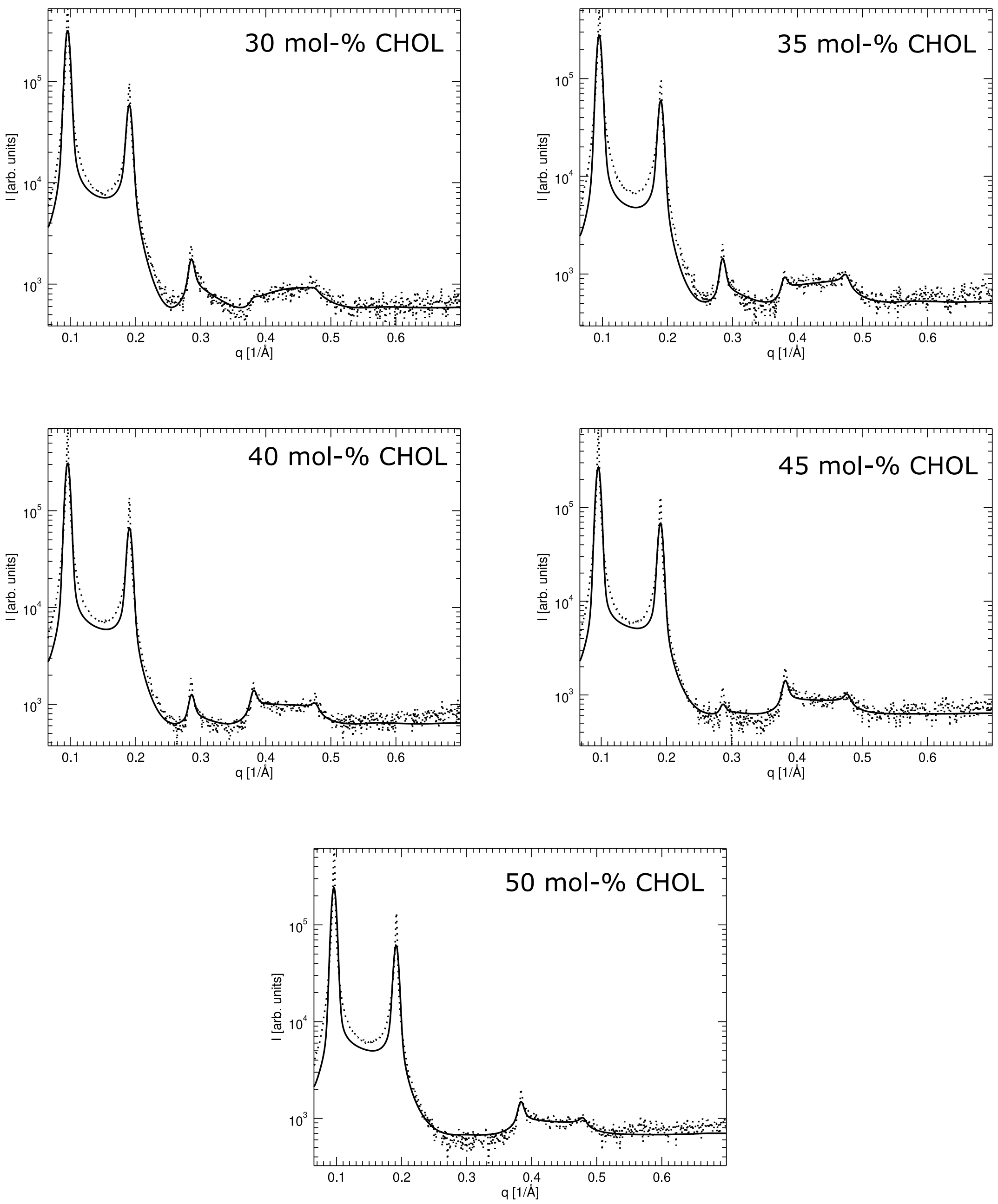

Figure S2: **Scattering intensities from X-ray scattering experiments with various concentrations of cholesterol.** Solid lines correspond to best fits using the SDP-GAP model. More data are shown in the previous figure.

2.2 Form Factors & Electron Density Profiles

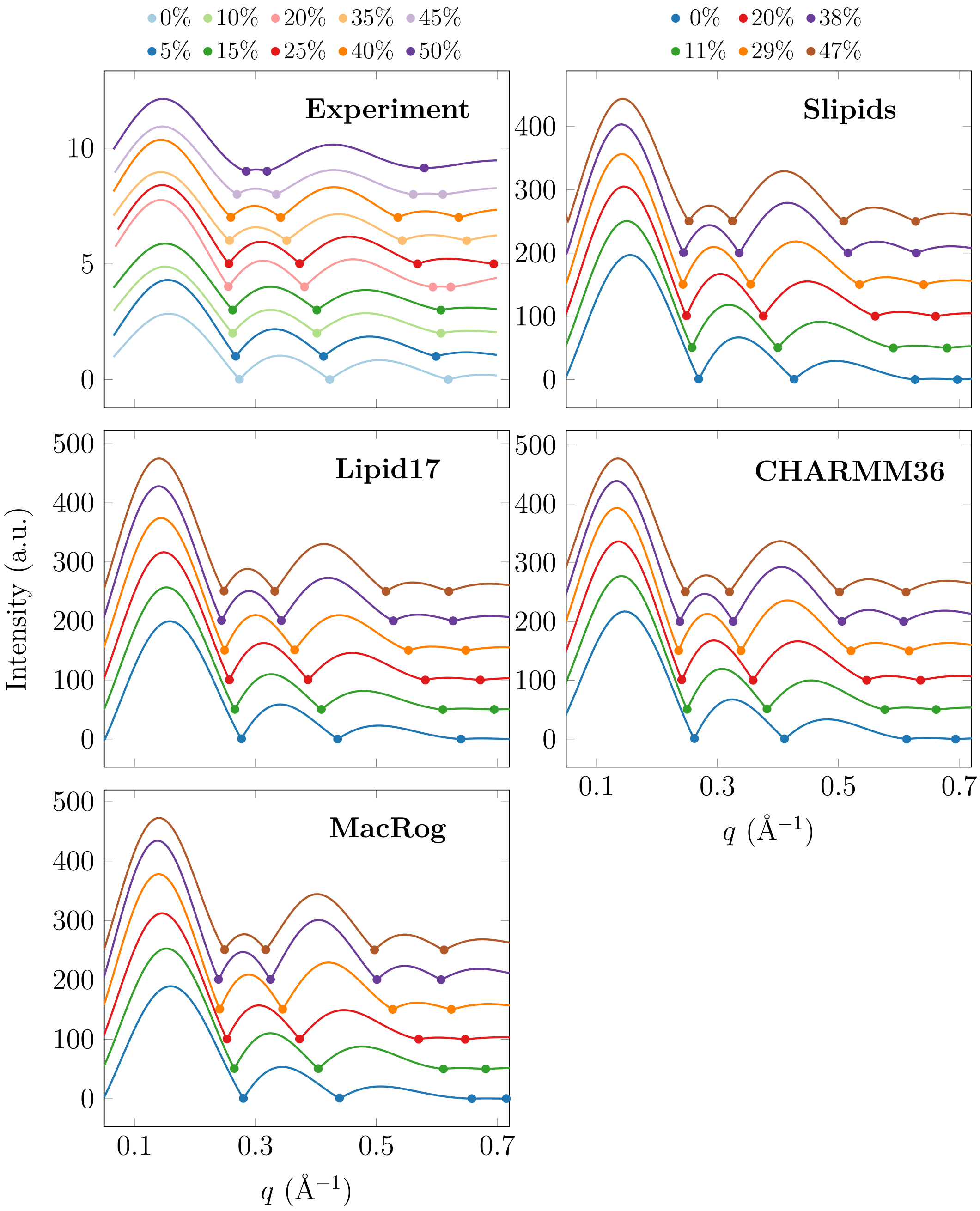

Figure S3: **Absolute values of X-ray scattering form factors.** Each of the profiles is shifted vertically with respect to the previous one, by 1 for the experimental profiles and by 50 for the computational ones. The minima are marked by filled circles to guide the eye. The numerical data for these plots can be found from the entries in the NMRlipids databank with the ID numbers listed in Table S2.

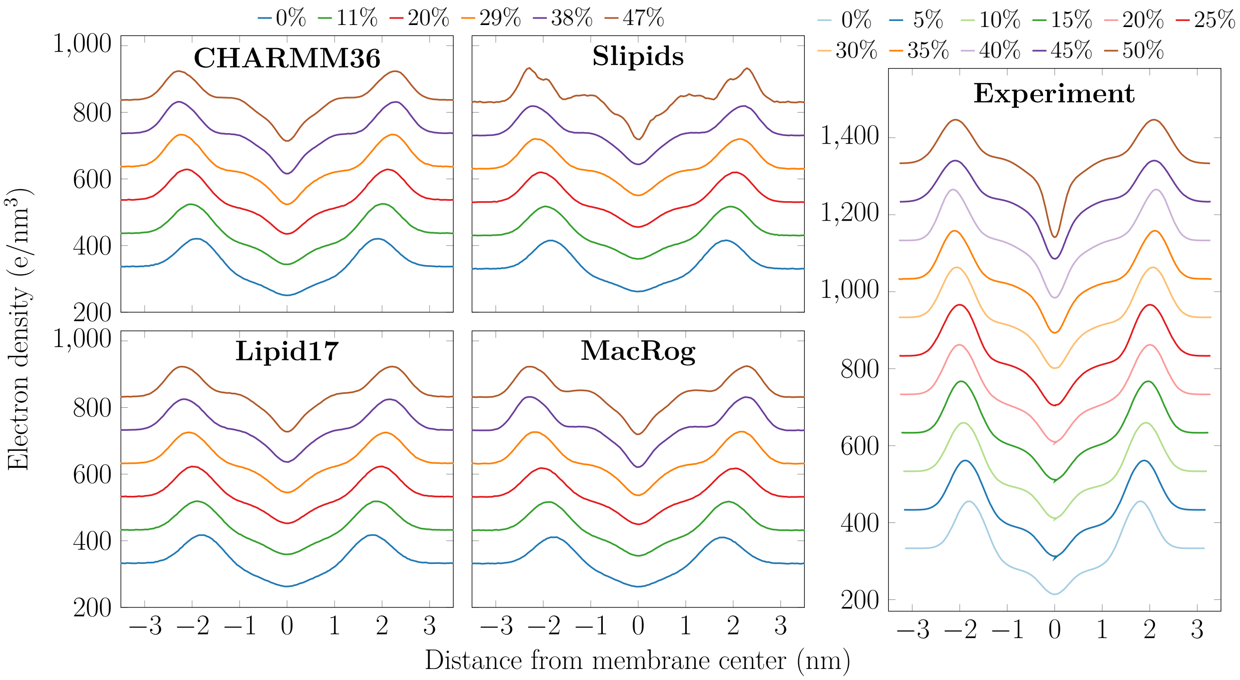

Figure S4: **Electron density profiles.** Each of the profiles is shifted vertically with respect to the previous one by 100 units. The numerical data for these plots can be found from the entries in the NMRlipids databank with the ID numbers listed in Table S2.

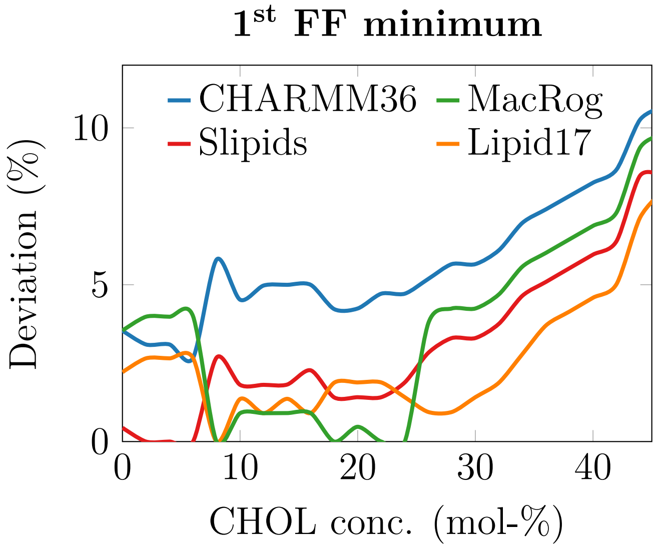

Figure S5: **Deviation of the first form factor minima.**

2.3 Order Parameters

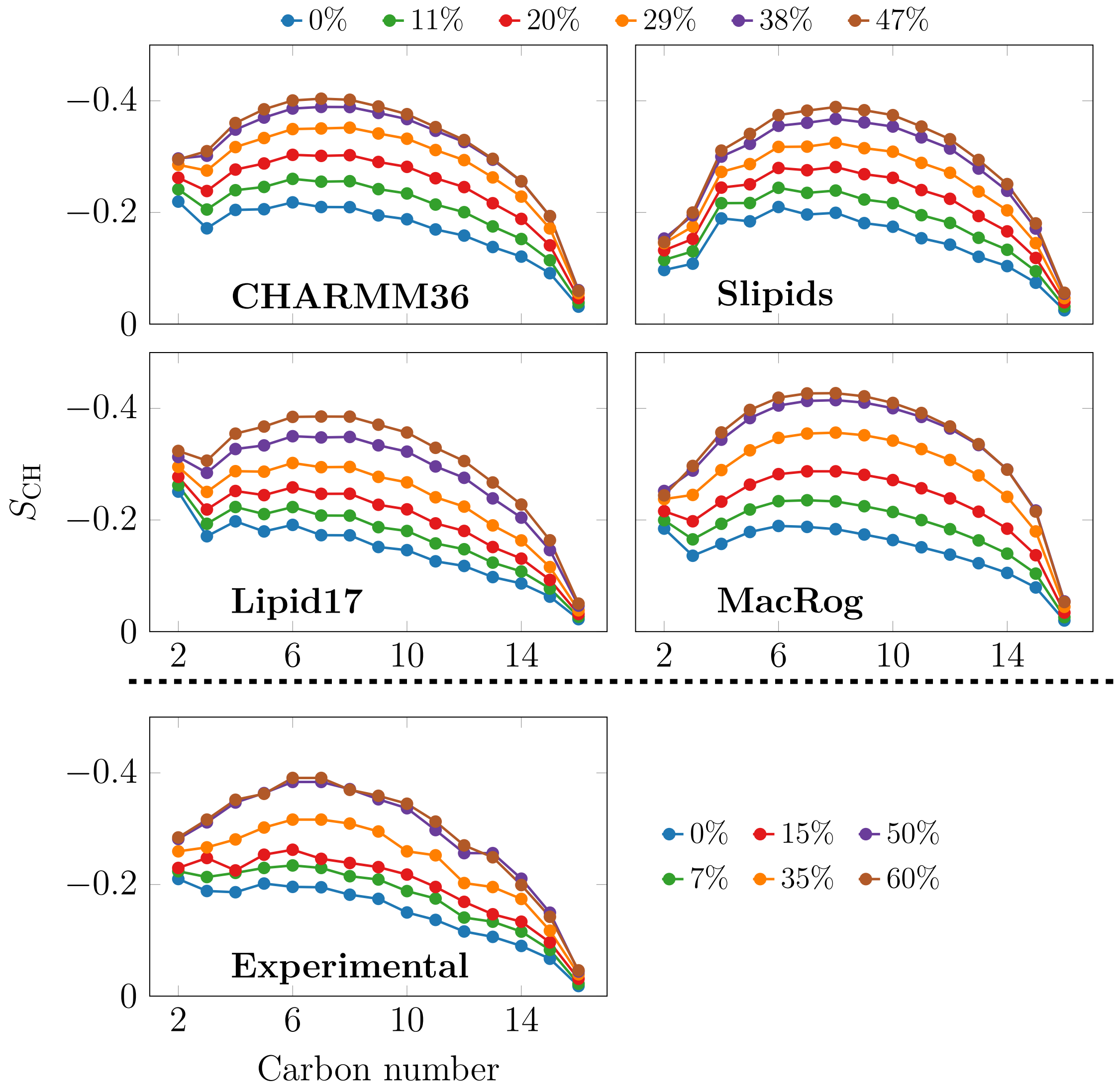

Figure S6: **Effect of cholesterol on the acyl chain order parameters of the POPC *sn*-1 (palmitate) chain.** The legend at the top corresponds to all simulations, and the one at the bottom to the experiments. Error bars in simulations are smaller than symbols. Error of the experimental data is estimated to be ±0.02.[S17] The numerical data for these plots can be found from the entries in the NMRlipids databank with the ID numbers listed in Table S2.

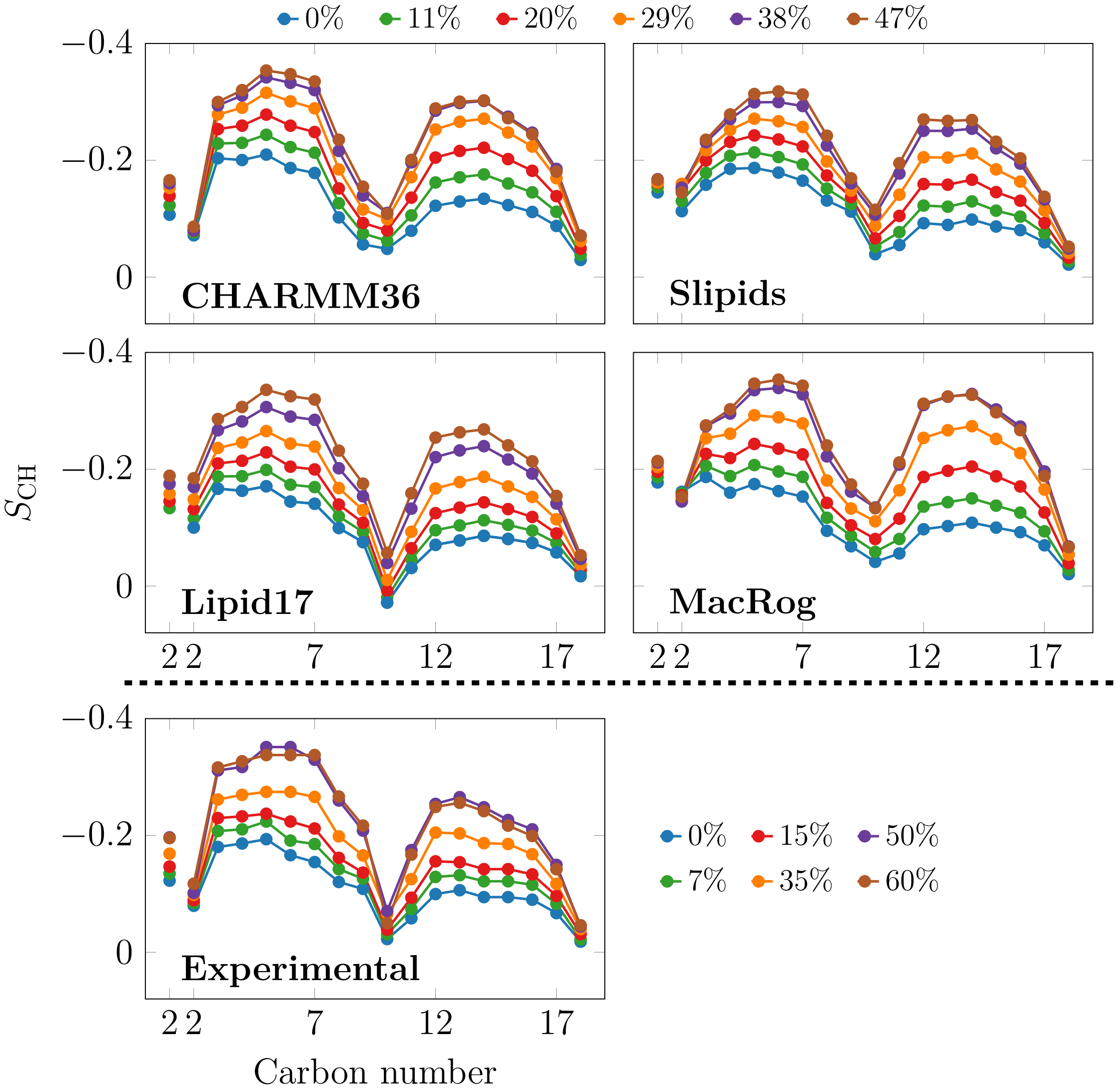

Figure S7: **Effect of cholesterol on the acyl chain order parameters of the POPC *sn*-2 (oleate) chain.** The legend at the top corresponds to all simulations, and the one at the bottom to the experiments. Since the order parameters measured for the two hydrogens bound to the C2 carbon differ, they are both shown in the plots. Stereospecific labeling is not done for these hydrogens but the one with larger value is shown first. Error bars in simulations are smaller than symbols. Error of the experimental data is estimated to be ±0.02.[S17] The numerical data for these plots can be found from the entries in the NMRlipids databank with the ID numbers listed in Table S2.

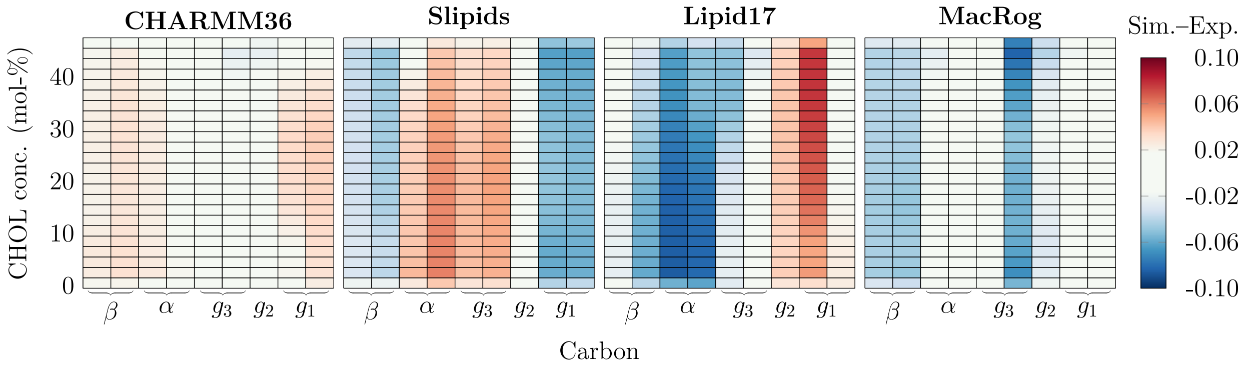

Figure S8: **The deviation of POPC head group parameters from experimental values as a function of CHOL concentration.**

2.4 Dynamic Properties

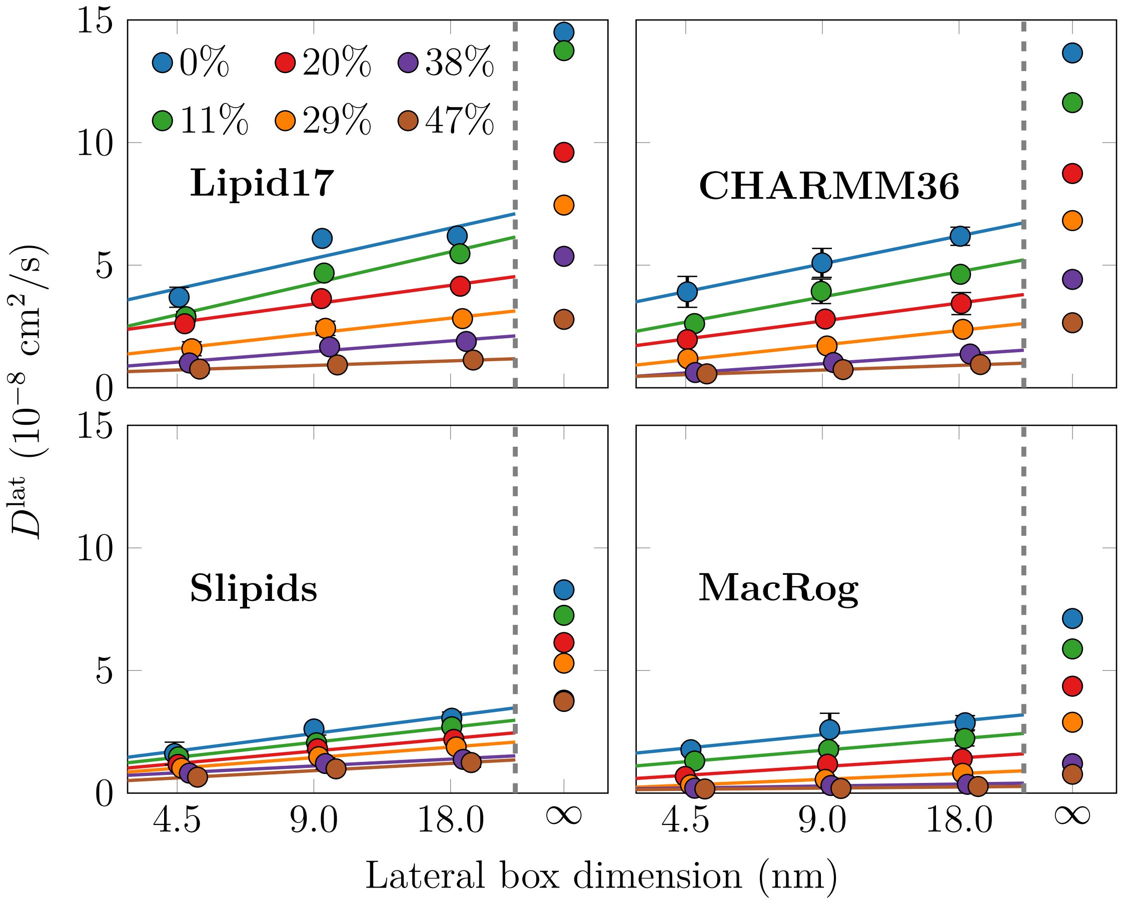

Figure S9: **Dependence of POPC lateral diffusion coefficients on the simulation box size.** The values calculated for the lipid centres of mass with `gmx msd` after eliminating leaflet drift. The values for the three system sizes are shown as markers together with fits of Eq. (**??**). The values extrapolated to infinite system sizes are also shown in the separate column.

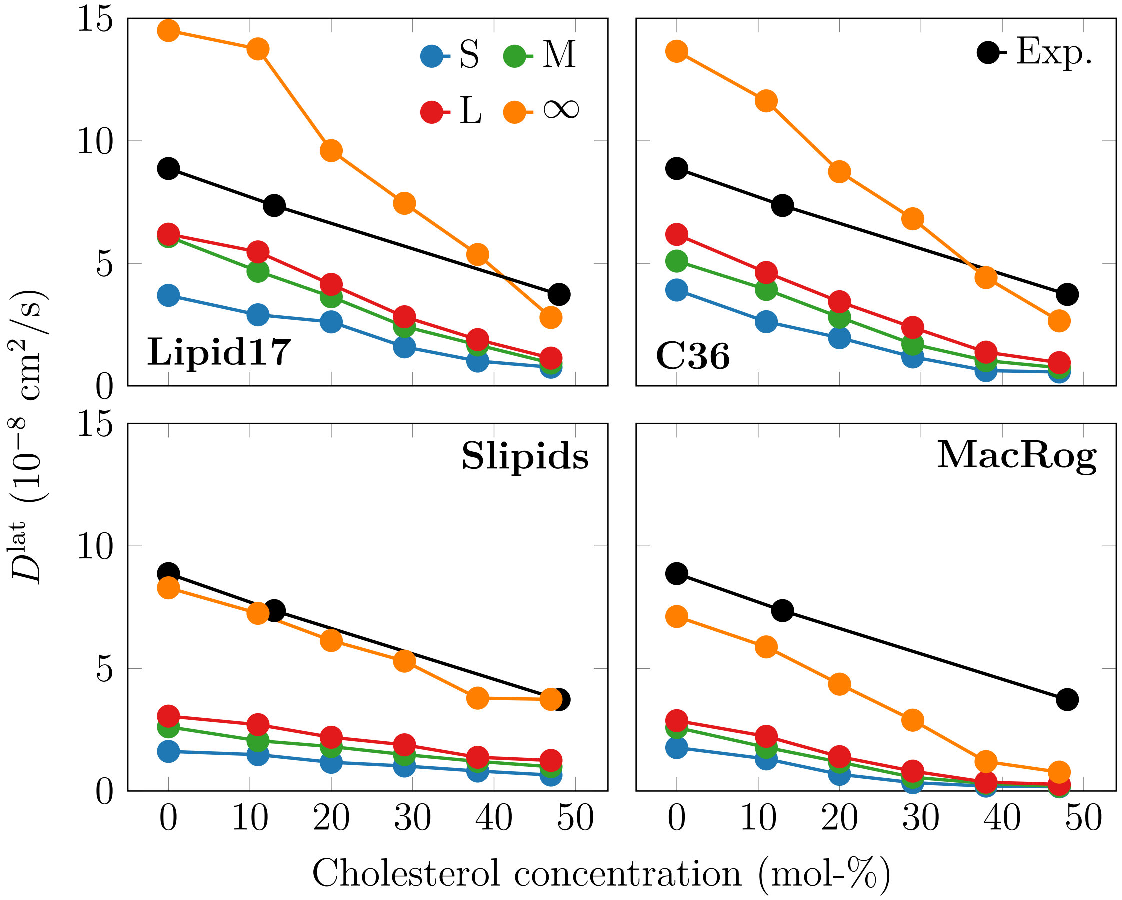

Figure S10: **Dependence of POPC lateral diffusion coefficients on cholesterol concentration.** Data are shown for all system sizes; small (S), medium (M), and large (L). The values extrapolated to infinity are shown as well (∞). Experimental data were measured at a hydration level of 55 wt-% of water,[S18,S19] whereas simulations have a hydration level of $\sim$54 wt-%.

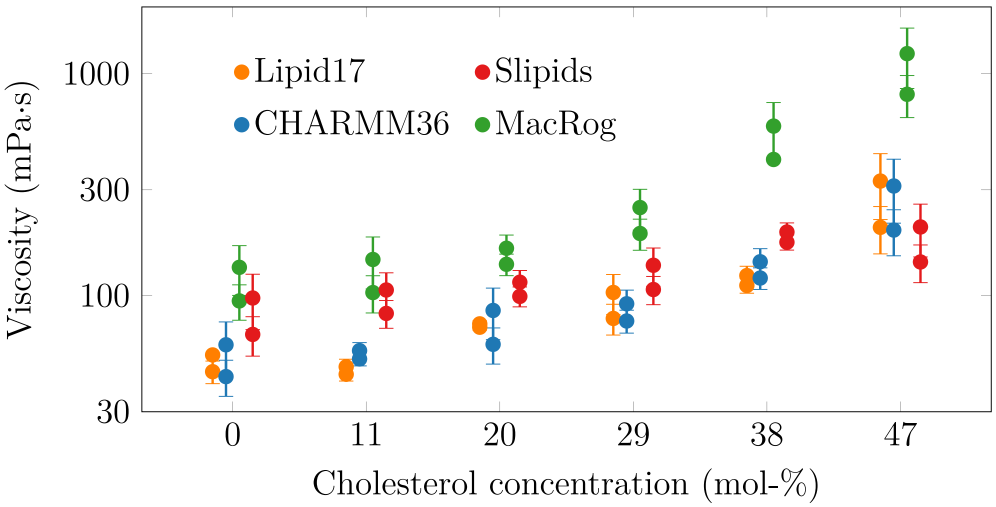

Figure S11: **Error estimation for viscosities.** The error in D_∞ was estimated by performing 10,000 fits of Eq. (**??**) with each D value assigned a random error (see the caption of Fig. **??** in the main text). These fits revealed a skewed Gaussian distribution of the viscosity values, which could be fitted by two Gaussians. The mean values and the corresponding standard deviations are shown here for all simulations. For some simulations, the distribution is close to unimodal and the two markers overlap, whereas for some they deviate significantly. The data for different force fields are slightly shifted horizonatally for readability.

2.5 Finite-Size Effects

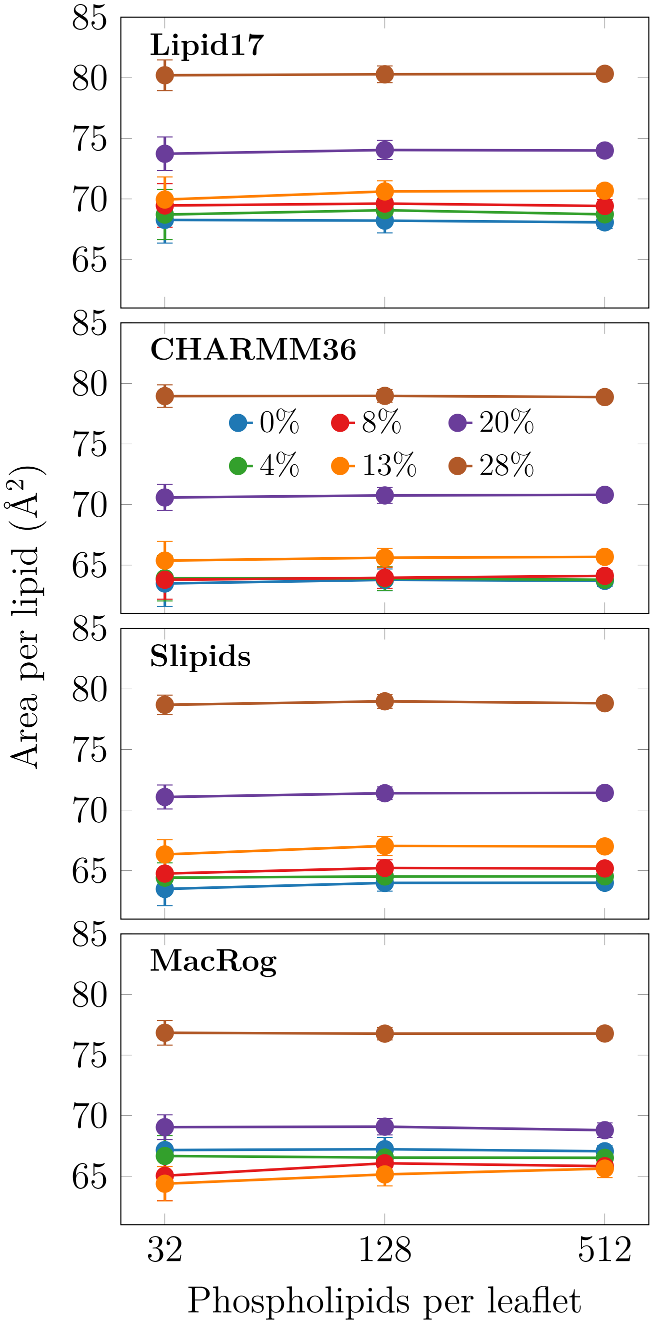

Figure S12: **Dependence of area per lipid on simulation size.** Area per lipid is calculated by dividing the box area by the number of lipids in one leaflet. Error bars show standard error extracted using block averaging in `gmx analyze`.

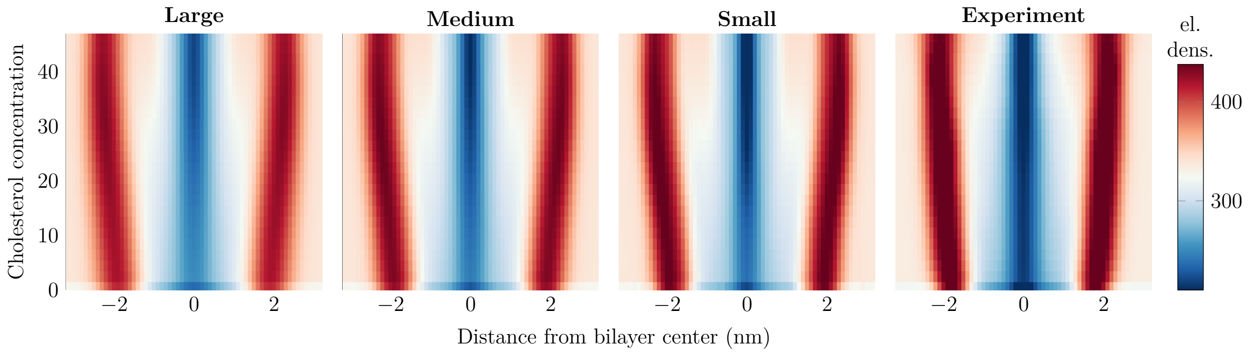

Figure S13: **Effect of system size on the density profiles.** Membrane undulations are larger in the larger systems, which leads to the smearing of the electron density profiles. Here, data are shown for CHARMM36 in the large (1024 POPC in total), medium (256 POPC in total), or small (64 POPC in total) systems. The experimental electron density profile is shown for comparison.

References

(S1) Hess, B.; Bekker, H.; Berendsen, H. J. C.; Fraaije, J. G. E. M. LINCS: A Linear Constraint Solver for Molecular Dynamics Simulations. *J. Comput. Chem.* **1997**, *18*, 1463–1472.

(S2) Hess, B. P-LINCS: A Parallel Linear Constraint Solver for Molecular Simulation. *J. Chem. Theory Comput.* **2008**, *4*, 116–122.

(S3) Kiirikki, A. M.; Antila, H. S.; Bort, L.; Buslaev, P.; Favela, F.; Ferreira, T. M.; Fuchs, P. F.; Garcia-Fandino, R.; Gushchin, I.; Kav, B.; Kula, P.; Kurki, M.; Kuzmin, A.; Madsen, J. J.; Miettinen, M. S.; Nencini, R.; Piggot, T.; Pineiro, A.; Samantray, S.; Suarez-Leston, F.; Ollila, O. H. S. NMRlipids Databank: Making Data-Driven Analyses of Membrane Properties Accessible for All. 2023; `https://doi.org/10.26434/chemrxiv-2023-jrpwm`.

(S4) Páll, S.; Hess, B. A Flexible Algorithm for Calculating Pair Interactions on SIMD Architectures. *Comput. Phys. Commun.* **2013**, *184*, 2641–2650.

(S5) Essman, U. L.; Perera, M. L.; Berkowitz, M. L.; Larden, T.; Lee, H.; Pedersen, L. G. A Smooth Particle Mesh Ewald Potential. *J. Chem. Phys.* **1995**, *103*, 8577–8592.

(S6) Darden, T.; York, D.; Pedersen, L. Particle Mesh Ewald: An N·log(N) Method for Ewald Sums in Large Systems. *J. Chem. Phys.* **1993**, *98*, 10089–10092.

(S7) Shirts, M. R.; Mobley, D. L.; Chodera, J. D.; Pande, V. S. Accurate and Efficient Corrections for Missing Dispersion Interactions in Molecular Simulations. *J. Phys. Chem. B* **2007**, *111*, 13052–13063.

(S8) Hoover, W. G. Canonical Dynamics: Equilibrium Phase-Space Distributions. *Phys. Rev. A* **1985**, *31*, 1695–1697.

(S9) Nose, S. A Molecular Dynamics Method for Simulations in the Canonical Ensemble. *Mol. Phys.* **1984**, *52*, 255–268.

(S10) Bussi, G.; Donadio, D.; Parrinello, M. Canonical Sampling Through Velocity Rescaling. *J. Chem. Phys.* **2007**, *126*.

(S11) Parrinello, M.; Rahman, A. Polymorphic Transitions in Single Crystals: A New Molecular Dynamics Method. *J. Appl. Phys.* **1981**, *52*, 7182–7190.

(S12) Berendsen, H. J.; Postma, J. v.; Van Gunsteren, W. F.; DiNola, A.; Haak, J. R. Molecular Dynamics With Coupling to an External Bath. *J. Chem. Phys.* **1984**, *81*, 3684–3690.

(S13) Durell, S. R.; Brooks, B. R.; Ben-Naim, A. Solvent-Induced Forces Between Two Hydrophilic Groups. *J. Phys. Chem.* **1994**, *98*, 2198–2202.

(S14) Jorgensen, W. L.; Chandrasekhar, J.; Madura, J. D.; Impey, R. W.; Klein, M. L. Comparison of Simple Potential Functions for Simulating Liquid Water. *J. Chem. Phys.* **1983**, *79*, 926–935.

(S15) Kulig, W.; Pasenkiewicz-Gierula, M.; Róg, T. *Cis* and *Trans* Unsaturated Phosphatidylcholine Bilayers: A Molecular Dynamics Simulation Study. *Chem. Phys. Lipids* **2016**, *195*, 12–20.

(S16) Milan Rodriguez, P.; Fuchs, P. F. MacRog Pure POPC MD Simulation (300 K - 500ns - 1 bar). 2020; `https://doi.org/10.5281/zenodo.3741793`.

(S17) Ollila, O. S.; Pabst, G. Atomistic Resolution Structure and Dynamics of Lipid Bilayers in Simulations and Experiments. *Biochim. Biophys. Acta* **2016**, *1858*, 2512–2528.

(S18) Filippov, A.; Orädd, G.; Lindblom, G. The Effect of Cholesterol on the Lateral Diffusion of Phospholipids in Oriented Bilayers. *Biophys. J.* **2003**, *84*, 3079–3086.

(S19) Filippov, A.; Orädd, G.; Lindblom, G. Influence of Cholesterol and Water Content on Phospholipid Lateral Diffusion in Bilayers. *Langmuir* **2003**, *19*, 6397–6400.
